# Psychometric Validation of the Hepatitis C Symptom and Impact Questionnaire (HCV-SIQv4) in a Diverse Sample of Adults with Chronic Hepatitis C Virus Infection Treated with an Interferon-free Simeprevir-containing Regimen

**DOI:** 10.36469/9675

**Published:** 2019-02-18

**Authors:** Andrew Trigg, Eric Chan, Helen Kitchen, Tom Willgoss, Kai Fai Ho, Renee Pierson, Jane Scott

**Affiliations:** 1 Formerly of DRG Abacus, Manchester, United Kingdom; 2 Janssen Global Services, LLC, Raritan, NJ, USA; 3 DRG Abacus, Manchester, United Kingdom; 4 STAT-TU Inc, Toronto, ON, Canada; 5 Janssen Global Services, LLC, High Wycombe, United Kingdom

**Keywords:** validation, psychometric, quality of life, patient reported outcomes, hepatitis

## Abstract

**Background:** Hepatitis C virus (HCV) infection and its treatments are associated with significant symptoms, side effects and impact on patients functioning. The Hepatitis C Symptom and Impact Questionnaire version 4 (HCV-SIQv4) was developed according to FDA Patient Reported Outcomes (PRO) Guidance, for evaluating chronic HCV infection and its treatment.

**Objectives:** This study evaluated the psychometric properties and clinically important change (CIC) thresholds of the measure.

**Methods:** PRO data were pooled from three Phase IIb and III trials evaluating interferon-free simeprevircontaining regimens for treatment of chronic HCV infection. Scale range adequacy, reliability, validity, responsiveness and CIC thresholds were assessed incorporating knowledge of the appropriate measurement model.

**Results:** Data from 437 patients were analyzed. Stage of liver disease was associated with symptom severity and functioning at baseline. Reliability was acceptable (test-retest ICC ≥0.7) for most scores except the Gastrointestinal and Integumentary domains. Convergent validity was observed between HCV-SIQv4 scores and concurrent measures of conceptual similarity. Greater symptom severity and worse impact scores were associated with liver cirrhosis, depression, severe fatigue and health limitations. Patients who achieved SVR12 had better outcomes than those failing to. HCV-SIQv4 symptom and domain scores were responsive to changes in health state (effect sizes ≥0.5). Exploratory thresholds for change in scores indicating a clinically important improvement and worsening were HCV-SIQv4 Overall Body System Score (BSS), 8 and 8; Constitutional BSS, 10 and 10; Gastrointestinal BSS, 5 and 5; Psychiatric BSS, 8 and 8; Neurocognitive BSS, 8 and 8; and Integumentary BSS, 5 and 5.

**Conclusions:** The HCV-SIQv4 offers reliable, responsive assessments within HCV clinical development. CIC thresholds are now available to aid score interpretation.

## Background

Hepatitis C Virus (HCV) infection, primarily transmitted via contact with blood, becomes chronic in approximately 75% of cases,^1,2^ estimated to affect 2.2 – 3.2 million persons in the United States and 2.8% of persons globally.^3,4^ Infected individuals are at a higher risk of liver cirrhosis and hepatocellular carcinoma, both associated with further complications and increased mortality.^5–7^

Although often characterized as asymptomatic,^8^ an estimated 25% of patients experience non-specific symptoms such as fatigue, nausea and musculoskeletal pain.^5,9–11^ Indeed, patients with chronic HCV infection experience impairments in several aspects of health-related quality of life (HRQoL) compared to a healthy population, including physical functioning, social functioning, work and recreational activities.^10,12,13^ Treatment-related side effects such as depression, fever and gastrointestinal complications are also a concern for patients, particularly those on interferon-based therapies, although these may also occur due to chronic HCV infection alone.^14–16^

It is therefore important to be able to monitor and evaluate the symptoms, impacts and side effects of chronic HCV and its treatment. Self-report via patient-reported outcome (PRO) measures is one way to assess this from a patient’s perspective. PROs administered within clinical studies must undergo psychometric validation, a cumulative process in which empirical evidence is accumulated to support the meaningfulness and appropriateness of their score inferences for the population of interest.^17–19^ Regulatory and professional bodies have published guidance and recommendations on the development, validation and use of PRO measures, including evidence for reliability, validity and ability to detect change.[@8927; ^20,21^ It is also paramount to define the level of change observed on PRO scores considered clinically important, to aid interpretation of treatment benefit.^22,23^

PROs developed specifically for HCV offer a targeted approach to capture the concepts relevant to a HCV population. The Hepatitis C Symptom and Impact Questionnaire version 4 (HCV SIQv4) is a HCV-specific measure measuring concepts identified as important by patients chronically infected with HCV,^9^ for which psychometric properties have not been reported to date. Two other HCV-specific PROs exist: the HCV- PRO^24^ and Chronic Liver Disease Questionnaire – Hepatitis C Version (CLDQ-HCV)^25^; however, neither comprehensively measures a number of symptoms and treatment-related side effects reported by patients with HCV^9^ including fever, nausea, constipation, diarrhea, dry or itchy skin, jaundice, dry mouth and loss of appetite amongst others.

The objectives of this study were 1) to evaluate the psychometric properties of the HCV-SIQv4, used to support study endpoints in interferon-free chronic HCV treatment trials; and 2) to identify thresholds for interpreting clinically important changes in the scores of this measure.

## Methods

### Data Sources

Individual patient-level data were obtained and pooled from three clinical studies conducted in the USA and Canada evaluating an interferon-free simeprevir-containing regimen for treatment of chronic HCV infection, in which all patients received treatment: IMPACT,^26^ OPTIMIST1^27^ and OPTIMIST2.^28^ Patients were both treatment-naïve and treatment-experienced at baseline, and varying in cirrhotic status, ensuring a diverse pooled sample. PRO data were collected between 2014 and 2015. Each study was approved by the institutional review board or independent ethics committee at each site, and all patients provided written informed consent prior to participation.

### Assessments

Demographic and disease characteristics were collected at baseline. Several PRO measures were completed by patients at each scheduled study visit, collected electronically via a touch-screen computer and self-administered. All patients completed English language versions of each PRO. The assessment schedule for the three clinical studies is provided in the Supplementary Appendix.

#### Hepatitis C Symptom and Impact Questionnaire Version 4 (HCV-SIQv4)

The HCV-SIQv4 is a self-administered 33-item questionnaire that asks respondents to rate the severity of symptoms associated with HCV or its treatment (29 items) and how symptoms impacted their daily life (3 items) “over the past 7 days including today.” The HCV-SIQv4 is typically completed in under 10 minutes. The 29 symptom-related items are of primary interest to this study.

The HCV-SIQv4 was developed over several stages of research and according to the FDA PRO guidance.^29^ Initial content was developed through qualitative input from patients with HCV in Canada, France, Germany and the US.^9^ In addition, targeted cognitive debriefing interviews were conducted to assess HCV patients’ understanding and relevance of a prior version, the HCV-SIQv3. This resulted in the addition of three new items assessing constipation, liver pain and jaundice and some minor formatting modifications, resulting in the HCV-SIQv4. The HCV-SIQv4 was translated into 14 languages using forward and backward translation and cognitive testing, according to industry standards.^30^

The scoring of the HCV-SIQv4 (Table 1) was derived based on an evaluation of the HCV-SIQv3. First, the verbatim symptom or side effect within each item was mapped to a MedDRA preferred term and associated MedDRA system organ class with clinical expert input. The MedDRA system organ class was used to inform the grouping of symptoms and side effects into scales. For example, the items “easily irritated,” “sad or depressed” and “worried or anxious” mapped to the MedDRA preferred terms “irritability,” “depressed mood” and “anxiety” of the “psychiatric” system organ class – this yielded the three-item psychiatric scale. Of the three additional items included within the HCV-SIQv4, those assessing constipation and liver pain were grouped within the gastrointestinal scale whilst the jaundice item was grouped within the integumentary scale, again based on MedDRA preferred terms.

In light of the above, the 29 symptom items of the HCV-SIQv4 are organized into six Body System Scores (BSS): Constitutional (CBSS), Gastrointestinal (GBSS), Psychiatric (PBSS), Neurocognitive (NBSS), Integumentary (IBSS) and Injection Site (ISBSS). An Overall BSS (OBSS) and Total Symptom Score (TSS) is also available, representing the total burden of chronic HCV infection, with alternative scoring excluding the Injection Site domain score available for therapies administered orally only (OBSS-IS and TSS-IS; Table 1). Although none of the studies from which data were extracted administered an injectable treatment, the injection site item, ISBSS, TSS and OBSS are included in this paper to confirm this item is behaving in the expected manner with the majority of responses to the injection site indicating no issues. All scoring options provide scores from 0 to 100; higher scores indicate greater symptom severity.

Notably, item 30 of the HCV-SIQv4, measuring health limitations, is also used within this analysis to group patients. The item is worded as follows: “Over the past 7 days, how much did your health limit you doing things you needed to do?” with four possible responses: “not at all limited,” “a little limited,” “somewhat limited” and “very limited.”

**Table 1.** HCV-SIQv4 Scoring Algorithms

**Scores**	**Item Number**	**Scoring algorithm**
**Total Symptom Score**	1–29	**Average of individual item scores**
**Total Symptom Score without Injection Site (TSS-IS)**	1–22, 24–29	Average of individual item scores except item #23
**Overall Body System Score**	-	**Average of Body System Scores**
		Constitutional Body System Score (CBSS)
		Gastrointestinal Body System Score (GBSS)
		Psychiatric Body System Score (PBSS)
		Neurocognitive Body System Score (NBSS)
		Integumentary Body System Score (IBSS)
		Injection Site Body System Score (ISBSS)
**Overall Body System Score without Injection Site (OBSS-IS)**	-	Average of all Body System Scores except ISBSS
**Body System Scores (BSS)**		
**Constitutional (CBSS)**		**Average of 7 item scores**
	1	Feverish (feeling hot, sweating or cold)
	2	Sore or achy muscles or joints
	3	Headache
	8	Shortness of breath
	9	Tiredness
	10	Physically weak
	27	Loss of appetite/did not feel like eating
**Gastrointestinal (GBSS)**		**Average of 9 item scores**
	4	Queasy or nauseous
	5	Stomach pain or cramps
	6	Pain or discomfort around your liver
	7	Constipation
	20	Pain or burning near anus
	24	Dry mouth
	26	Diarrhea (very loose or liquid stools)
	28	Things taste bad or had little flavor
**Psychiatric (PBSS)**		**Average of 3 item scores**
	11	Easily irritated
	12	Sad or depressed
	13	Worried or anxious
**Neurocognitive (NBSS)**		**Average of 5 item scores**
	14	Trouble remembering things
	15	Trouble thinking clearly or concentrating
	16	Problems getting to sleep or staying asleep
	22	Feeling faint or dizzy
	25	Ringing or buzzing sound in ears
**Integumentary (IBSS)**		**Average of 5 item scores**
	17	Dry or itchy skin
	18	Tender or irritated skin
	19	Jaundice (yellowish skin or eyes)
	21	Hair loss
	29	Your hair or nails look or feel bad (dry, dull, break easily)
**Injection Site reactions (ISBSS)**		**Single item score**
	23	Soreness or swelling where medicine was injected

HCV-SIQv4: Hepatitis C Symptom and Impact Questionnaire version 4

Item scores ranging from 0-100 obtained through multiplying response by (100/number of response options – 1).

Responses for items 1 to 25: Not at all (0); A little (1); Somewhat (2); Very (3); Extremely (4). Responses for items 26 & 27: 0 days (0); 1-2 days (1); 3-4 days (2); 5-6 days (3); Every day (4). Responses for items 28 & 29: No (0); Yes (1).

#### Other PRO Assessments

Each trial within this analysis also administered the Fatigue Severity Scale (FSS),^31^ Center for Epidemiologic Studies Depression Scale (CES-D)^32^ and the EuroQol 5-Dimension 5-Level Questionnaire (EQ-5D-5L).^33^ The FSS has evidence to support its reliability and validity in chronic HCV patients and previous usage in HCV clinical trials.^34–36^ A total score is obtained by averaging the nine items; higher scores indicate greater severity of fatigue.

The CES-D, a 20-item self-administered PRO measuring depressive symptoms, has been psychometrically evaluated in a HCV population, supporting its reliability and validity.^37,38^ A total score is calculated as the sum of the items; higher scores indicate greater depressive symptom severity.

The EQ-5D-5L is a generic measure of overall health status assessed by five items corresponding to five health dimensions (mobility, self-care, usual activities, pain/discomfort and anxiety/depression). An index score for health utility assessment^39^ and a Visual Analogue Scale (VAS) measuring overall perceived health status were obtained, where higher scores indicate better health status. The EQ-5D-5L has been administered in numerous HCV clinical studies and possesses strong measurement properties.^33,36,40–43^

### Statistical and Psychometric Analyses

In line with FDA PRO guidance,^29^ all statistical procedures undertaken were specified *a priori* in a statistical analysis plan. Any missing data at the item-level were handled in accordance with the scoring algorithms of each PRO measure. Missing data at the score-level were not imputed. All analyses were conducted using SAS software (Statistical Analysis System, Version 9.3). All tests for statistical significance were assessed at the 0.05 level. The majority of psychometric analyses conducted at a single point in time used Week 4 data, as this was the first assessment common to all trials where patients were receiving treatment and thus able to experience treatment-related side effects.

The validation of the HCV-SIQv4 was conducted in recognition that the instrument, designed to incorporate the various symptoms and side effects experienced across all patients and treatments, would be unlikely to conform to effect indicator measurement models. Effect indicator models assume that items are a manifestation of the latent construct one aims to measure, where a change in the construct leads to changes in all items; thus, effect indicators are commonly viewed as interchangeable.^44^ The latent construct is often defined by the common variance between items using methods such as factor analysis or item response theory; high correlations among effect indicators are thus expected.^45,46^

In contrast, it is recognized that symptom or side-effect scales are often best represented by causal indicator models,^45,46^ where items contribute a unique aspect of the construct and thus are not necessarily correlated. Causal indicators correspond to the theoretical definition of a latent variable and are assumed to cause changes in perceived health.^44^ The homogeneity of causal indicators is not assumed, and approaches based on this assumption such as factor analysis, item response theory and internal consistency reliability are consequently of limited value.^46–48^ Dangers of applying such methods suitable for effect indicator models to measures that are in fact based on causal indicators include the unnecessary deletion of “poorly-fitting” items, which are in fact an essential and defining feature of the construct one intends to measure.^45^ The capitalization on correlations between side-effects due to the administration of a specific treatment rather than the presence of an influential latent construct driving responses is also likely.^45^ Additionally, floor effects are not seen as a weakness in causal indicators, since it is recognized that many patients would not experience certain symptoms which nonetheless would form a vital contribution towards perceived health.^46^ Instead, in such cases it is most important to comprehensively assess the symptoms and side-effects providing unique contributions to the constructs of interest, which patients deem relevant and important in relation to their experience as demonstrated in the aforementioned qualitative research.^9^

Notably, often a criterion or “gold standard” measure is recommended to evaluate the adequacy of casual indicators and are necessary for identification purposes in structural equation models.^44^ Although a suitable criterion measure was not available for all the symptoms and side-effects captured by the HCV-SIQv4 (a common occurrence, as recognized by the FDA20), a pragmatic approach using convergent PRO instruments, clinical characteristics and clinician-reported adverse events was employed. Criterion measures can also inform weighting procedures for scoring, often recommended for causal indicator models.^49^ However, in the absence of a theory or data to guide weighting, average-based scoring (implying equal weighting) was retained.^50^ Such an approach may also facilitate the use of scores within applied research.^51^

#### Analysis Populations

All analyses were conducted on the intent-to-treat (ITT) populations defined for each study^26–28^; however, data from one study site (n=3 patients) were excluded from analyses due to non-compliance with study PRO administration protocols. Sub-populations according to clinical characteristics were also analyzed to compare performance in diverse patient sub-groups.

#### Descriptive Summaries

Demographic and disease characteristics collected at baseline were descriptively summarized. HCV-SIQv4 scores and compliance (the proportion of subjects with at least one non-missing response) at each scheduled visit were summarized.

#### Distribution of Scores

The frequencies of endorsed responses to each HCV-SIQv4 item was summarized at each time point. As an interpretative guideline, a floor or ceiling effect was considered to be present if >20% of responses (or >80% for dichotomous responses) to an item were at the lowest or highest level, respectively. Floor and ceiling effects were also assessed for HCV-SIQv4 scores, considered to be present if >20% of scores were at 0 or 100, in line with recommended guidelines within physical therapy.^52^ While floor effects were expected given the presence of causal indicators, ceiling effects were considered indicative of insufficient scale range.

#### Reliability

Test-retest reliability in stable patients was evaluated for all HCV-SIQv4 scores by calculating the intraclass correlation coefficient (ICC; 2,1 variant used)^53^ between scores at Week 1 and Week 2, where the least change was expected. Patients were defined as stable based on the absence of adverse events (AEs) relating to any of the HCV-SIQv4 symptom items, and was restricted to data from the OPTIMIST1 and OPTIMIST2 trials due to assessment timings (Supplementary Appendix). An ICC value of >0.70 was considered evidence of acceptable test-retest reliability.^54^ Test-retest reliability was also evaluated at the item level, using Cohen’s weighted kappa for polytomous items and a simple kappa for dichotomous items, using the following magnitudes of agreement: poor (<0.4), good (0.4 to 0.75) and excellent (>0.75).^55^

#### Construct Validity

Construct validity was assessed in the form of concurrent and known groups validity. Concurrent validity was assessed at Week 4 by testing hypothesized relationships between HCV-SIQv4 scores and the other administered PRO scores measuring similar constructs: the EQ-5D-5L Index, EQ-5D-5L VAS, CES-D total score and FSS total score. All hypothesized correlations were based upon the degree of conceptual overlap and prior experience with the HCV-SIQv3. The TSS-IS and OBSS-IS were also expected to be correlated >0.6 with the FSS total score, CES-D total score and EQ-5D-5L Index as all measure a symptom and health limitations-type construct. Additionally, the TSS-IS and OBSS-IS was hypothesized to be correlated >0.5 with the EQ-5D-5L VAS, as this targets more general aspects of HRQoL. Hypotheses were also made regarding four of the HCV- SIQv4 BSS scoring options based on conceptual overlap with other measures: CBSS (>0.5 with FSS, CES-D and EQ-5D-5L Index), GBSS (>0.5 with EQ-5D-5L Index), PBSS (>0.5 with CES-D and EQ-5D-5L Index) and NBSS (>0.5 with CES-D). As no concurrent assessments measured integumentary symptoms or injection- site reactions, concurrent validity was not assessed for the IBSS and ISBSS.

Known groups validity, the ability of HCV-SIQv4 scores to differentiate between known-groups hypothesized to differ on the concepts they measured, was assessed by comparing mean scores to a reference category using two-sample t-tests and between-group effect sizes. Several hypotheses were made based on extant research and past experience with the HCV-SIQv3. Older patients are more likely to have moderate or severe HCV compared to younger patients (reported on one study as 41.1 vs 49.5 years respectively, p=0.003^56^ and thus expected to report a greater severity of symptoms, due to HCV; thus, scores were compared between patients <50 years (reference category) and patients ≥50 years. Female patients report experiencing more severe symptoms, particularly psychiatric symptoms^37^; thus, scores were compared between male (reference category) and female patients. Severity and risk of developing symptoms during treatment were expected to be greater for patients who are obese,^57^ thus scores were compared between patients considered non-obese (<25 kg/m2) (reference category) and patients considered overweight (25 to <30 kg/m2) and patients considered obese (≥30 kg/ m2), according to current guidance. Patients with significant depressive symptomatology were also expected to experience greater symptom severity^37^; thus, scores were compared between patients with no depressive symptoms (CES-D score <16) (reference category), patients with subthreshold depression symptoms (CES-D score 16 to 22) and patients with greater depression risk (CES-D score 23-60).^58^ Patients with greater fatigue severity based on patients described as normal fatigue levels (FSS scores 1-3 (reference category), fatigued (FSS score 3-<4)^59^ and severe fatigue (FSS score 4-7) were expected to experience greater HCV symptom severity.^35^ The normal range was defined based on the mean plus one standard deviation of scores in healthy individuals.^60^ Additionally, patients with cognitive impairment,^61^ defined as normal (reference category) or impaired according to age matched norms for CogState tests, were expected to experience greater HCV symptom severity. Based on the authors’ past experience, patients with higher overall levels of health limitations (HCV-SIQv4 item 30) or problems doing usual activities (EQ-5D-5L item 3) were expected to have lower HCV-SIQv4 symptom scores, reflecting greater severity. Finally, it was hypothesized that patients with Sustained Virologic Response at Follow-Up Week 12 (SVR12) would have scores indicating significantly better health than those without SVR12.^36^ Specifics of each known group, including reference categories, are provided in Table 2.

**Table 2.** Known Groups Validity of HCV-SIQv4 OBSS-IS

**Known Groups**	**n**	**Mean**	**Effect Size**
**Age**			
<50 (ref)	68	12.6	-
≥50	362	12.1	0.04
**Sex**			
Male (ref)	261	11.2	-
Female	169	**13.7**	0.20
**BMI**			
<25 kg/m2 (ref)	120	11.8	-
25 to <30 kg/m2	154	11.8	0.00
≥30 kg/m2	156	13.0	0.10
**Depressive Symptoms**			
0 to 15 CES-D score (ref)	318	7.9	-
16 to 22 CES-D score	52	**22.7**	1.44
23 to 60 CES-D score	44	**30.3**	2.32
**Fatigue Severity**			
0 to <3 FSS score (ref)	224	7.2	-
3 to <4 FSS score	78	**13.2**	0.62
≥4 FSS score	118	**20.8**	1.23
**Health limitations**			
Not at all (ref)	274	7.1	-
A little	116	**18.6**	1.18
Somewhat/Very	40	**28.8**	2.03
**Usual Activities**			
No problems (ref)	288	7.9	-
Slight problems	79	**20.0**	1.21
Moderate/Severe/Unable to do	50	**24.6**	1.46
**Stage of liver disease**			
No cirrhosis (ref)	293	12.1	-
Compensated cirrhosis	95	**17.7**	0.42
Decompensated cirrhosis	39	**26.6**	1.01
SVR12*			
No (ref)	36	13.1	-
Yes	384	10.6	0.18
**Cognitive status**			
Normal (ref)	19	24.7	-
Impaired	20	28.5	0.23

* Defined as HCV RNA <25 IU/mL at Follow-up Week 12

Values in bold indicate that a hypothesized significant (p<0.05) known-group effect was observed. Patients were assigned to each known-group at Baseline, with the exception of health limitations (Week 4), usual activities (Week 4) and SVR12 (Follow Up Week 12). Analysis of PRO scores was conducted at Week 4 for all known groups analyses except stage of liver disease (Baseline), SVR12 (Follow-up Week 12) and cognitive status (Baseline). Impaired cognitive status was defined according to the Cogstate, a performance-based measure of cognitive functioning [65] administered within the IMPACT study only.

CES-D: Center for Epidemiologic Studies Depression Scale; FSS: Fatigue Severity Scale; HCV-SIQv4: Hepatitis C Symptom and Impact Questionnaire; OBSS-IS: Overall Body System Score excluding Injection Site; PRO: Patient Reported Outcomes; SVR 12: Sustained Virologic Response at Follow-Up Week 12

#### Responsiveness

Responsiveness, or ability to detect change, was assessed for each HCV-SIQv4 score by observing the change scores from Baseline to last study visit in patients classified into “no change”, “improved” and “worsened” groups based on a recommended^62^ approach of multiple health state anchors: EQ-5D-5L VAS (improved: ≥8 point increase, worsened: ≥8 point decrease^63^), FSS (improved: ≥1 point increase, worsened: ≥1 point decrease35), HCV-SIQv4 Health Limitations (improved: ≥1 response increase, worsened: ≥1 response decrease) and EQ- 5D-5L Usual Activities (improved: ≥1 response increase, worsened: ≥1 response decrease). Responsiveness to SVR12 (improvement only) and viral relapse at Follow-up Week 12 (worsening only) was also assessed.^36^ Additionally, responsiveness to AE reports was assessed at the item level for the HCV-SIQv4. Responsiveness was assessed using a within-group effect size in line with FDA guidance,^29^ where an effect size of 0.8 was considered large, 0.5 considered moderate, and 0.2 considered small.^64^ Responsiveness between-groups was also assessed using two-sample t-tests to determine whether the mean change scores for the “improved” and “worsened” groups were significantly different from the mean change scores for the “no change” group. Each responsiveness analysis was only performed if there were ≥30 patients in each group.

#### Clinically Important Change Thresholds

The median change in HCV-SIQv4 scores in each no change, improved and worsened groups defined for the responsiveness analyses was interpreted to determine preliminary thresholds for change that can be considered clinically important for a patient. The median was chosen based on the skewed distribution of score changes. Note that all responsiveness groups, according to each anchor, were assessed in conjunction. In addition to theoretical justification of the relationship between each anchor and HCV-SIQv4 score, empirical justification was informed by the within-group effect sizes calculated for responsiveness, where larger effect sizes in the expected direction were indicative of better anchor performance. The relative effect sizes observed for each anchor-scale combination guided the relative subjective weighting of CIC estimates when forming recommendations.

## Results

### Descriptive Analysis

Demographic and clinical characteristics of the overall pooled population (N=437) at Baseline are shown in Table 3. Baseline HCV-SIQv4 scores of the overall pooled population are shown in Table 4. Subjects’ compliance with the HCV-SIQv4 during the three clinical studies was very high (>95.9%) at all study visits.

### HCV-SIQv4 Psychometric properties

A summary of the HCV-SIQv4’s key psychometric properties is provided in Table 4.

#### Scale Range Adequacy

All response options (except “extremely” on Item 23) were endorsed by one or more subjects at some point during the study. Floor effects (indicating no experience of a symptom) were observed for all HCV-SIQv4 symptom items 1–28 at Baseline and across all visits, as expected. Floor effects were observed for Item 29 (Hair or nails look or feel bad) at Week 1 only (86.0%). No ceiling effects were observed for any HCV- SIQv4 item at any time point. At the score level, floor effects were observed for the OBSS, OBSS-IS, TSS and TSS-ISS at Follow-up Week 24 only (22.5% for all scores) and for all individual Body System Scores at all time points. No ceiling effects were observed for any HCV-SIQv4 score.

**Table 3.** Demographic and Clinical Characteristics for Overall Pooled Population at Baseline

**Characteristic**	**N=437**
**Age (years)**	
Mean (SD)	55.2 (9.34)
Range	19, 75
**Sex, n (%)**	
Female	173 (39.6%)
Male	264 (60.4%)
**Race, n (%)**	
Asian	6 (1.4%)
Black	74 (16.9%)
Caucasian	351 (80.3%)
Other	2 (0.5%)
Missing	4 (0.9%)
**Body Mass Index (kg/m2)**	
Mean (SD)	28.78 (6.105)
Range	16.5, 56.4
**HCV Genotype, n (%)**	
Genotype 1a	316 (72.3%)
Genotype 1b	120 (27.5%)
Genotype 4	1 (0.2%)
**IL28B Genotype, n (%)**	
CC	112 (25.6%)
CT	245 (56.1%)
TT	79 (18.1%)
Missing	1 (0.2%)
**Liver Cirrhosis, n (%)**	
No	301 (68.9%)
Compensated	95 (21.7%)
Decompensated	40 (9.2%)
Missing	1 (0.2%)
**Time Since Diagnosis (years, N=435)**	
Mean (SD)	11.67 (8.298)
Range	0.2, 41.5
**Treatment History, n (%)**	
Experienced	156 (35.7%)
Naïve	281 (64.3%)

SD: standard deviation

**Table 4.** Comparison of Key Psychometric Properties and Clinically Important Change Thresholds for PRO Measures

**Score**	**Score at Baseline**	**Test-retest Reliability**	**Concurrent Validity**				**Clinically important Change**
	Mean (SD)	ICC in stable subjects (95% CI)	Pearson's correlation between scores				Change in score indicating a clinically important improvement/worsening
**HCV-SIQv4**			**FSS**	**CES-D**	**EQ-5D-5L VAS**	**EQ-5D-5L Index**	
TSS	13.4 (12.8)	0.84 (0.794, 0.879	-	-	-	-	-8/8
TSS-IS	13.9 (13.2)	0.84 (0.792, 0.877)	0.56^b^	0.68^a^	-0.64^a^	-0.61^a^	-8/8
OBSS	12.4 (11.9)	0.85 (0.802, 0.884)	-	-	-	-	-8/8
OBSS-IS	14.6 (13.7)	0.85 (0.804, 0.884)	0.57^b^	0.71^a^	-0.64^a^	-0.62^a^	-8/8
CBSS	16.4 (16.5)	0.86 (0.821, 0.886)	0.59^a^	0.59^a^	-0.61	-0.57^a^	-10/10
GBSS	9.2 (12.6)	0.69 (0.620, 0.741)	0.39	0.50	-0.49	-0.48^b^	-5/5
PBSS	18.6 (20.6)	0.75 (0.704, 0.795)	0.51	0.74^a^	-0.57	-0.57^a^	-8/8
NBSS	17.2 (18.0)	0.84 (0.804, 0.868)	0.50	0.64^a^	-0.58	-0.57	-8/8
IBSS	11.8 (15.2)	0.60 (0.516, 0.663)	0.26	0.33	-0.33	-0.30	-5/5

Dashes (-) indicate property not assessed or was unable to meaningfully evaluate.

^a^ indicates hypothesis for concurrent validity met, ^b^ indicates hypothesis not met, other correlations for which no hypotheses were made are retained for completeness.

TSS: Total Symptom Score; TSS-IS: Total Symptom Score excluding Injection Site; OBSS: Overall Body System Score; OBSS-IS: Overall Body System Score excluding Injection Site; CBSS: Constitutional Body System Score; GBSS: Gastrointestinal Body System Score; PBSS: Psychiatric Body System Score; NBSS: Neurocognitive Body System Score; IBSS: Integumentary Body System Score; ISBSS: Injection Site Body System Score; FSS: Fatigue Severity Scale; CES-D: Center for Epidemiologic Studies Depression Scale; EQ-5D-5L: EuroQol 5-dimension questionnaire 5-levels; ICC: Intraclass correlation coefficient; CI: confidence interval

#### Reliability

Acceptable test-retest reliability (ICC>0.7) was observed in stable patients for the following HCV-SIQv4 scores: TSS, TSS-IS, OBSS, OBSS-IS, CBSS, PBSS and NBSS. The OBSS-IS in particular retained this acceptable test- retest reliability across all analysis sub-populations. ICC values for the GBSS and IBSS were 0.69 (95% CI 0.62-0.74) and 0.59 (0.52-0.66), respectively. At the item level, all but four of the 29 HCV-SIQv4 symptom items failed to demonstrate a good magnitude of agreement (kappa≥0.4) in stable patients: Item 19 (jaundice) (kappa=0.39), Item 20 (pain or burning near anus) (kappa=0.34) and Item 28 (Things taste bad or had little flavor) (kappa=0.38).

#### Construct Validity

Correlations between PRO scores, detailing which concurrent validity hypotheses were met, are provided in Table 4. Importantly, the PBSS and NBSS were correlated with the CES-D total score to the hypothesized degree, and the CBSS was correlated with the FSS total score to the hypothesized degree. Known-groups comparisons of HCV-SIQv4 OBSS-IS scores are provided in Table 2. All individual BSS scores were significantly higher in patients with greater health limitations and more advanced liver disease, as hypothesized.

#### Responsiveness

**Figure 1.**
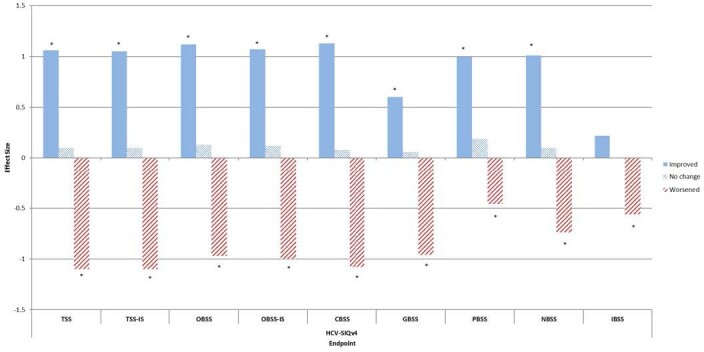
Responsiveness of HCV-SIQv4 Scores from Baseline to Last Study Visit, by Change in Health Limitations over this Time *Indicates significant difference between Improved/Worsened and No Change groups. Positive and negative effect sizes represent and improvement and worsening in score, respectively. Effect sizes for improvement on all HCV-SIQv4 scores except IBSS surpassed the “moderate” threshold; effect sizes for worsening on all HCV- SIQv4 scores except PBSS and ISBSS surpassed the “moderate” threshold.

Responsiveness to improvement and worsening in health state as measured by HCV-SIQv4 Item 30 (Health limitations) is shown in Figure 1.

OBSS-IS scores were also responsive to improvement and worsening in health state as defined by EQ-5D-5L VAS (effect sizes of 0.55 and 0.45); FSS Total Score (effect sizes of 0.93 and 0.55); and SVR12 (effect size of 0.35, worsening not assessed). The responsiveness of OBSS-IS scores was broadly consistent across analysis sub-populations, including non-cirrhotic patients (median change in OBSS-IS of -7.63 and 12.44 for respective improvement and worsening as measured by HCV-SIQv4 Item 30, compared to -9.19 and 9.60 in the pooled population). HCV-SIQv4 items were responsive to reports of related adverse events (effect sizes 0.29–1.30). [Supplemental figures show the effect sizes observed for all scores based on all anchors.]

### Clinically Important Change Thresholds

Based on the effect sizes observed in responsiveness analyses (see supplemental figures), the HCV SIQv4 Item 30 (Health limitations) anchor was most influential when triangulating estimates to arrive at recommended thresholds. Given the comparatively low effect sizes observed for anchors based on SVR12 and viral relapse, CIC estimates obtained through these approaches carried relatively little weight during triangulation. Suggested clinically important change thresholds for improvement and worsening are provided in Table 4. In practice, sensitivity analyses based on the range of CIC estimates in Supplementary Table 1 can be applied. The OBSS- IS 8-point threshold was consistent across analysis subpopulations except in genotype 1b or 4 or obese subjects where 7 points may be more appropriate. Thresholds for worsening were not attainable for subjects with decompensated cirrhosis, as too few of these patients worsened in health state.

## Discussion

This is the first study investigating the psychometric properties of the HCV-SIQv4 in a large, diverse chronic HCV sample. Importantly, the evaluation of psychometric properties was conducted in light of the causal indicator models underlying the questionnaire.

The HCV-SIQv4 offers multiple scoring options for researchers. In addition to the OBSS and TSS scoring systems providing an assessment of overall burden due to HCV-related symptoms and side effects, the individual BSS scores enable focused measurement on specific classes of symptoms. Alternatively, the individual items of the HCV-SIQv4 could be used to measure safety within a trial as a form of adverse event reporting. All response options (except “extremely” on Item 23 [soreness or swelling at injection site], as expected) were endorsed at least once, supporting the relevance of all response options of the HCV-SIQv4. No ceiling effects were observed, and floor effects were as expected given that many patients would not experience certain symptoms.

The stability of HCV-SIQv4 scores over time was also supported; test-retest reliability was acceptable for all scores except the GBSS and IBSS. All items within the IBSS, however, did demonstrate acceptable stability as measured by kappa coefficients. The OBSS-IS in particular was stable over time across multiple analysis sub- populations. However, test-retest analyses are best performed in accordance with an appropriate indicator of stability in health status e.g. a Patient Global Impression of Change (PGI-C).^48^ As no suitable measure was included within the clinical studies, a less-rigorous approach in the form of spontaneously reported AEs was employed, although the observed results remain promising.

The concurrent validity of the HCV-SIQv4 is strongly supported by the study findings, with the majority of hypothesized correlations met. Correlations with the FSS and CES-D indicate that HCV-SIQv4 scores are valid measures of fatigue and depression whilst also yielding additional, important information related to HCV and treatment-specific symptoms.

The known-groups validity of the HCV-SIQv4 is largely supported by the study results, demonstrating that each score can distinguish key sub-groups known to affect the relevant constructs of interest. The hypothesized differences in scores for known-groups according to age, sex and cognitive status were rarely observed in terms of statistical significance. However, hypothesized significant differences were consistently observed according to depressive symptoms, fatigue severity, health limitations, usual activities and stage of liver disease. Although patients with SVR12 did have scores indicating better health than those without, such effects were not significant, perhaps due to the limited number of patients failing to achieve SVR12.

A key objective of this validation study was to explore the responsiveness of HCV-SIQv4 scores to changes in health state. Most HCV-SIQv4 symptom items and scores, especially the TSS and OBSS options, demonstrated strong responsiveness to improvement and worsening in health state. Responsiveness of symptom items to AE reports supports the purpose of the HCV-SIQv4 as a measure intended to capture treatment side effects. Promisingly, the observed responsiveness to improvement within the non-cirrhotic sub-population demonstrated that the HCV-SIQv4 is sensitive to improvements in health status even in those patients at the lowest level of severity.

It is imperative that PRO measures are evidenced as valid, reliable, responsive assessments when intended to support clinical trial endpoints. However, of equal importance is the ability to interpret score changes that occur longitudinally in order to understand the potential clinical benefit of the treatment under investigation. To this end, thresholds for clinically important change scores have been recommended for each HCV-SIQv4 score, facilitating interpretation in future studies. Thresholds for the OBSS-IS in particular were largely consistent across analysis sub-populations, except in patients with genotype 1b or 4 infection and obese patients; however, this should be interpreted with caution due to small sample sizes within these groups.

Limitations of this study are acknowledged. As previously noted, no patient-reported indicator of stability or change was administered in the clinical studies, limiting the robustness of the test-retest and responsiveness analyses, and suggested clinically important change thresholds. Also, the fact that no injectable treatments were administered in any of the studies means that the analyses conducted on the ISBSS can be considered exploratory, although this item did behave in the manner expected. While the specific effect of each causal indicator on its latent construct can be estimated through structural equation modelling approaches, it was not possible to fit such models due to a lack of suitable criterion measures and subsequent underidentification.^44^ Although the HCV-SIQv4 Item 30 provides a general overview of HCV-related health limitations, in practice two measures are required to assess the fit of structural equation models, and ones more directly targeted to each score’s content are preferable. Therefore, future work should confirm the HCV-SIQv4 domains and scoring through this approach if suitable criterion measures can be identified, including the possibility of weight-based scoring which may confer advantages over the current approach.^49^ Additionally, sample sizes for some responsiveness analyses and subgroup comparisons were not always sufficient to allow interpretation of results e.g. clinically meaningful worsening in subjects with decompensated cirrhosis. Finally, the sample primarily comprised patients who were genotype 1a or 1b infected; further evaluation in other genotype and treatment populations is advised.

## Conclusions

This study, conducted in line with regulatory and professional guidance, provides evidence to support the psychometric strength of the HCV-SIQv4 and provides further information on its utility in a chronic HCV population. The HCV-SIQv4 OBSS-IS score in particular has demonstrated reliability, validity and ability to detect change across several demographic and clinical subgroups and may be the most suitable scale of the HCV-SIQv4 to measure symptoms specifically related to HCV and its treatment. Thresholds for clinically important change are suggested, aiding the interpretability of scores obtained in future clinical studies. In light of the limitations of this study, other researchers are encouraged to document the psychometric properties of the HCV-SIQv4 in future studies within the HCV infected population in order to further confirm the psychometric properties of this PRO.

**Table:** Abbreviations

AE	Adverse events
BSS	Body System Score
CBSS	Constitutional Body System Score
CES-D	Center for Epidemiologic Studies Depression Scale
CIC	Clinically important change
CLDQ-HCV	Chronic Liver Disease Questionnaire – Hepatitis C Version
EQ-5D-5L	EuroQol 5-Dimension 5-Level Questionnaire
FSS	Fatigue Severity Scale
GBSS	Gastrointestinal Body System Score
HCV	Hepatitis C virus
HCV-SIQv4	Hepatitis C Symptom and Impact Questionnaire version 4
HRQoL	Health-related quality of life
IBSS	Integumentary Body System Score
ICC	Intraclass correlation coefficient
IS	Injection site
ISBSS	Injection Site Body System Score
ITT	Intent-to-treat
NBSS	Neurocognitive Body System Score
OBSS	Overall Body System Score
PBSS	Psychiatric Body System Score
PGI-C	Patient Global Impression of Change
PRO	Patient Reported Outcomes
SAS	Statistical Analysis System
SVR12	Sustained Virologic Response at Follow-up Week 12
TSS	Total Symptom Score
VAS	Visual Analogue Scale

## Funding

This study was funded by Janssen Global Services.

## Authors’ contributions

JS, TW, KFH and HK designed the study and statistical analysis plan. KFH performed the statistical analysis. JS, TW, KFH, HK, AT, RP and EC interpreted findings and participated in the study coordination. All authors read and approved the final manuscript.

## Availability of data and material

All data generated or analyzed during this study will be made available upon reasonable request.

## Competing interests

At the time of study completion AT, TW and HK were employed by DRG Abacus, a healthcare outcomes agency that consults with various pharmaceutical companies. DRG Abacus received fees for the analyses and reporting of the data in this manuscript. RP, EC and JS are employees of Janssen Global Services who funded the study and own stocks in Johnson and Johnson. KFH received consultancy fees for conducting analyses and reviewing the manuscript.

[Bibr ref-8972]

## Supplementary Material

Supplementary Content
