# Supplementary material for: Psychometric Validation of the Hepatitis C Symptom and Impact Questionnaire (HCV-SIQv4) in a Diverse Sample of Adults with Chronic Hepatitis C Virus Infection Treated with an Interferon-free Simeprevir-containing Regimen
Source: J Health Econ Outcomes Res. 2019 Feb 18;6(2):1–19. doi: 10.36469/9675 (PMC7299472; doi:10.36469/9675)
Supplement: Supplementary Content [file jheor_2019_6_2_9675_34287.pdf]

JHEOR Journal of Health Economics and Outcomes Research

OPEN ACCESS

Supplementary Online Content

Trigg A, Chan E, Kitchen H. Psychometric validation of the hepatitis C symptom and impact questionnaire (HCV-SIQv4) in a diverse sample of adults with chronic hepatitis C virus infection treated with an interferon-free simeprevir-containing regimen. *JHEOR*. 2019;6(2):1-19.

Supplemental Table 1. Comparison of Change from Baseline in HCV-SIQv4 Symptom Scores Between Subjects with Worsened Health Limitations, Improved Health Limitations and Subjects with No Change in Health Limitations
Supplemental Figure 1. Responsiveness of HCV-SIQv4 Scores from Baseline to Last Study Visit, by Change in EQ-5D-5L VAS over this Time
Supplemental Figure 2. Responsiveness of HCV-SIQv4 Scores from Baseline to Last Study Visit, by Change in Fatigue Severity Scale over this Time
Supplemental Figure 3. Responsiveness of HCV-SIQv4 Scores from Baseline to Last Study Visit, by Change in EQ-5D-5L Usual Activity Score over this Time
Supplemental Figure 4. Responsiveness of HCV-SIQv4 Scores from Baseline to Last Study Visit, by Adverse Events over this Time
Supplemental Figure 5. Responsiveness of HCV-SIQv4 Scores from Baseline to Last Study Visit, by change in SVR12 over this Time
Supplemental Figure 6. Responsiveness of HCV-SIQv4 Scores from Baseline to Last Study Visit, by Viral Relapse over this Time

This supplementary material has been provided by the authors to give readers additional information about their work.

Supplemental Table 1. Comparison of Change from Baseline in HCV-SIQv4 Symptom Scores Between Subjects with Worsened Health Limitations, Improved Health Limitations and Subjects with No Change in Health Limitations

	**EQ-5D-5L VAS**			**FSS Total Score**			**HCV-SIQv4 Item 30 Health Limitations**			**EQ-5D-5L Usual Activities**		
	**Improved**	**No Change**	**Worsened**	**Improved**	**No Change**	**Worsened**	**Improved**	**No Change**	**Worsened**	**Improved**	**No Change**	**Worsened**
N	140	201	61	110	217	75	81	265	63	69	268	65
Change at LSV												
HCV-SIQv4 Scores												
TSS												
**Median**	**-4.31**	**0.00**	**2.59**	**-6.90**	**0.00**	**2.59**	**-8.62**	**-0.86**	**9.48**	**-8.62**	**0.00**	**3.45**
Mean	-5.86	-0.05	4.89	-8.84	-0.09	6.14	-10.46	-1.04	10.06	-9.82	-0.56	4.54
SD	13.37	9.37	12.93	11.89	9.12	13.69	14.29	8.12	12.68	15.03	8.49	15.80
TSS-IS[a]												
**Median**	**-4.46**	**0.00**	**2.68**	**-7.14**	**0.00**	**2.68**	**-8.93**	**-0.89**	**9.82**	**-8.93**	**0.00**	**3.57**
Mean	-6.00	-0.02	5.06	-9.07	-0.06	6.36	-10.68	-1.06	10.45	-10.03	-0.57	4.77
SD	13.76	9.65	13.39	12.21	9.40	14.18	14.71	8.39	13.12	15.53	8.78	16.20
OBSS												
**Median**	**-4.59**	**0.00**	**3.15**	**-6.92**	**-0.48**	**2.78**	**-7.86**	**-0.60**	**8.00**	**-9.17**	**-0.46**	**2.30**
Mean	-5.79	-0.40	4.19	-8.64	-0.34	5.19	-10.17	-1.14	8.24	-9.72	-0.70	3.46
SD	12.19	8.77	11.44	10.93	8.29	12.48	13.13	7.49	11.71	13.60	7.72	14.65
OBSS-IS[a]												
**Median**	**-5.34**	**0.00**	**3.78**	**-7.53**	**-0.58**	**3.33**	**-9.19**	**-0.70**	**9.60**	**-9.15**	**-0.55**	**2.76**
Mean	-6.59	-0.28	5.03	-9.91	-0.22	6.23	-11.35	-1.28	10.04	-10.87	-0.81	4.53
SD	14.11	10.21	13.72	12.49	9.65	14.98	15.06	8.86	13.92	16.06	9.18	16.64

[a]Excludes score from Item #23 "Soreness or swelling where medicine was injected"

Change groups: Improved (≤ -1), No Change (0), Worsened (≥ 1)

LSV: Last Scheduled Visit; HCV-SIQv4: Hepatitis C Virus Symptom and Impact Questionnaire version 4; TSS: Total Symptom Score; TSS-IS: Total Symptom Score excluding Injection Site; OBSS: Overall Body System Score; OBSS-IS: Overall Body System Score excluding Injection Site; CBSS: Constitutional Body System Score; GBSS: Gastrointestinal Body System Score; PBSS: Psychiatric Body System Score; NBSS: Neurocognitive Body System Score; IBSS: Integumentary Body System Score; ISBSS: Injection Site Body System Score; FSS: Fatigue Severity Scale; EQ-5D-5L: EuroQol 5-dimension questionnaire-5 levels

Supplemental Table 1. Comparison of Change from Baseline in HCV-SIQv4 Symptom Scores Between Subjects with Worsened Health Limitations, Improved Health Limitations and Subjects with No Change in Health Limitations - continued

	**EQ-5D-5L VAS**			**FSS Total Score**			**HCV-SIQv4 Item 30 Health Limitations**			**EQ-5D-5L Usual Activities**		
	**Improved**	**No Change**	**Worsened**	**Improved**	**No Change**	**Worsened**	**Improved**	**No Change**	**Worsened**	**Improved**	**No Change**	**Worsened**
N	140	201	61	110	217	75	81	265	63	69	268	65
CBSS												
**Median**	**-3.57**	**0.00**	**7.14**	**-8.93**	**0.00**	**10.71**	**-10.71**	**0.00**	**10.71**	**-10.71**	**0.00**	**7.14**
Mean	-7.83	-0.16	8.26	-11.49	-0.92	11.19	-14.90	-1.11	14.34	-14.49	-0.99	9.84
SD	18.22	12.95	18.75	16.87	12.01	19.18	17.81	11.48	19.09	19.865	11.53	21.71
GBSS												
**Median**	**0.00**	**0.00**	**3.13**	**-3.13**	**0.00**	**3.13**	**-3.13**	**0.00**	**6.25**	**-3.13**	**0.00**	**3.13**
Mean	-3.08	0.58	2.61	-5.17	0.58	3.83	-6.60	-0.65	9.52	-5.53	-0.02	3.56
SD	14.29	9.99	12.55	13.72	10.80	11.51	15.82	9.05	12.91	16.19	8.95	16.54
PBSS												
**Median**	**-8.33**	**0.00**	**0.00**	**-16.67**	**0.00**	**0.00**	**-16.67**	**0.00**	**8.33**	**-16.67**	**0.00**	**8.33**
Mean	-10.83	-2.49	4.10	-16.14	-1.96	5.78	-17.08	-3.21	7.94	-18.24	-3.14	5.13
SD	20.96	17.26	19.93	19.27	15.31	23.29	21.36	15.48	23.78	23.67	15.03	24.19
NBSS												
**Median**	**-5.00**	**0.00**	**0.00**	**-10.00**	**0.00**	**0.00**	**-10.00**	**0.00**	**5.00**	**-10.00**	**0.00**	**5.00**
Mean	-8.32	-0.85	5.25	-12.14	-0.92	6.93	-14.69	-1.45	9.84	-14.06	-1.31	4.69
SD	19.08	13.18	15.287	17.23	12.90	17.45	19.60	12.59	15.91	21.53	11.10	21.99
IBSS												
**Median**	**0.00**	**0.00**	**0.00**	**0.00**	**0.00**	**0.00**	**0.00**	**0.00**	**5.00**	**0.00**	**0.00**	**0.00**
Mean	-2.89	1.54	4.92	-4.64	2.12	3.40	-3.46	0.04	8.57	-2.03	1.42	-0.54
SD	18.65	13.76	20.38	18.58	15.05	17.82	20.57	14.01	20.15	20.53	15.68	17.28

[a]Excludes score from Item #23 “Soreness or swelling where medicine was injected”

Change groups: Improved (≤ -1), No Change (0), Worsened (≥ 1)

LSV: Last Scheduled Visit; HCV-SIQv4: Hepatitis C Virus Symptom and Impact Questionnaire version 4; TSS: Total Symptom Score; TSS-IS: Total Symptom Score excluding Injection Site; OBSS: Overall Body System Score; OBSS-IS: Overall Body System Score excluding Injection Site; CBSS: Constitutional Body System Score; GBSS: Gastrointestinal Body System Score; PBSS: Psychiatric Body System Score; NBSS: Neurocognitive Body System Score; IBSS: Integumentary Body System Score; ISBSS: Injection Site Body System Score; FSS: Fatigue Severity Scale; EQ-5D-5L: EuroQol 5-dimension questionnaire-5 levels

Supplemental Figure 1. Responsiveness of HCV-SIQv4 Scores from Baseline to Last Study Visit, by Change in EQ-5D-5L VAS over this Time

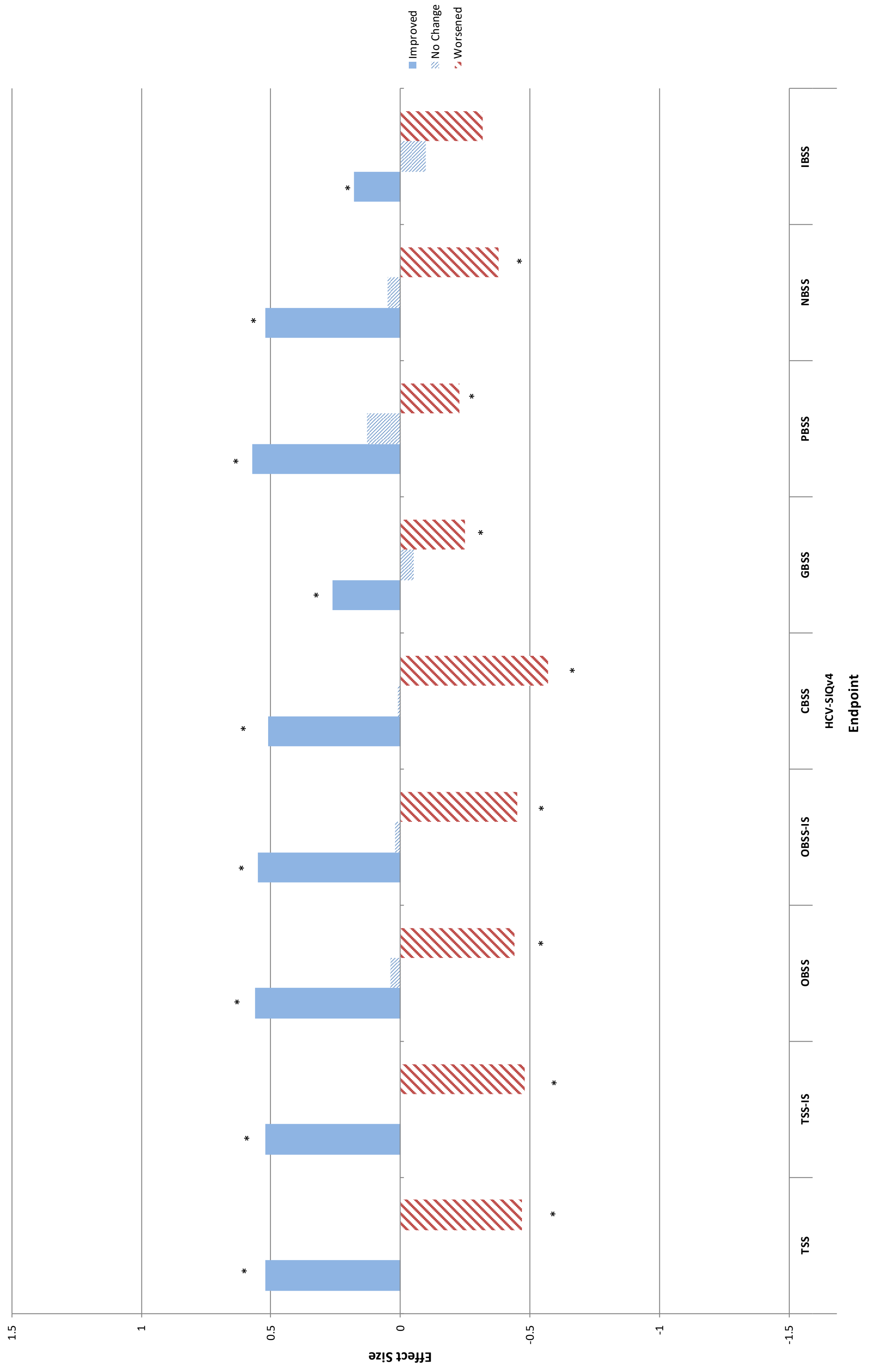

* Denotes p<0.05 as assessed by two-sample t-test between groups

Supplemental Figure 2. Responsiveness of HCV-SIQv4 Scores from Baseline to Last Study Visit, by Change in Fatigue Severity Scale over this Time

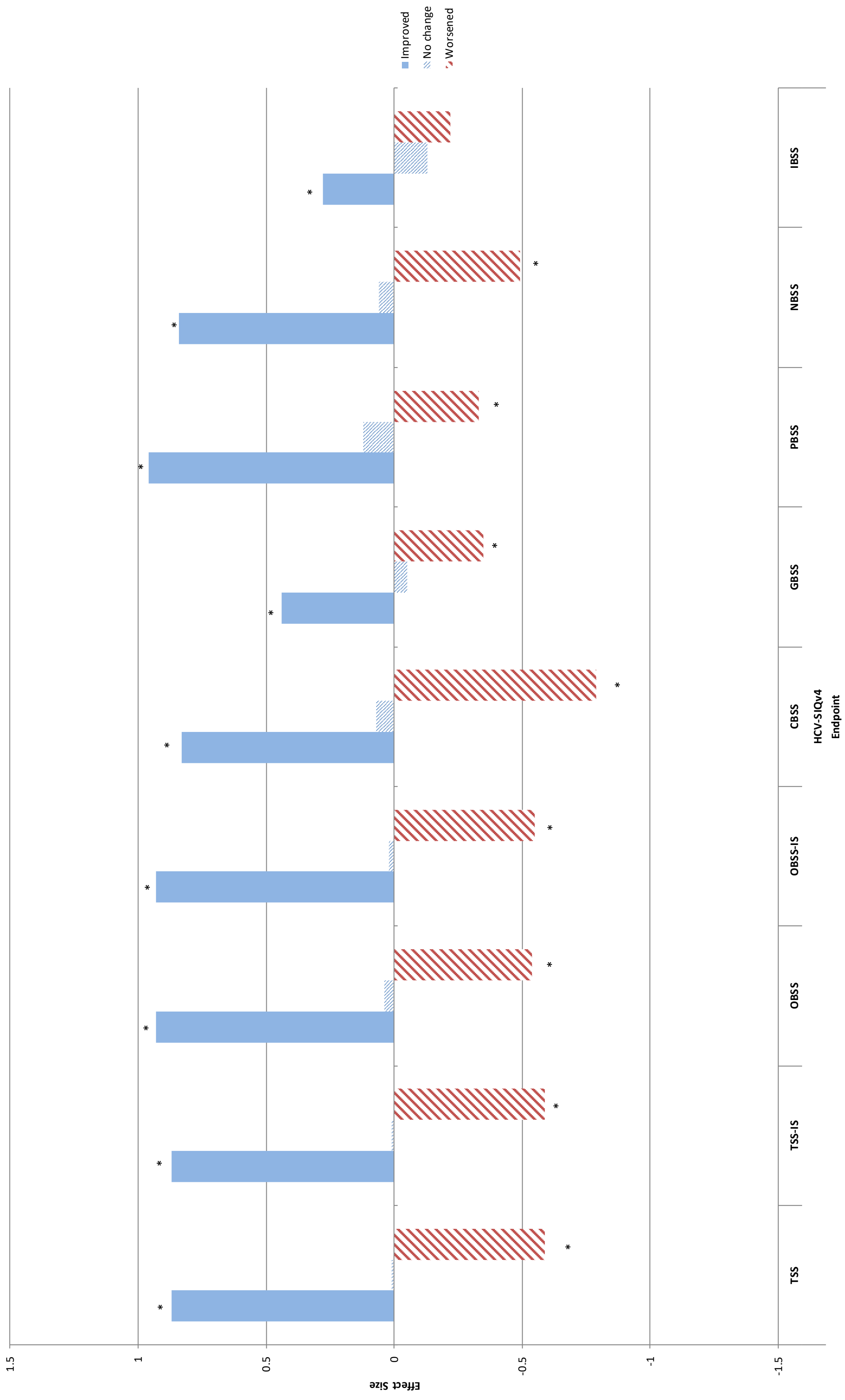

* Denotes p<0.05 as assessed by two-sample t-test between groups

Supplemental Figure 3. Responsiveness of HCV-SIQv4 Scores from Baseline to Last Study Visit, by Change in EQ-5D-5L Usual Activity Score over this Time

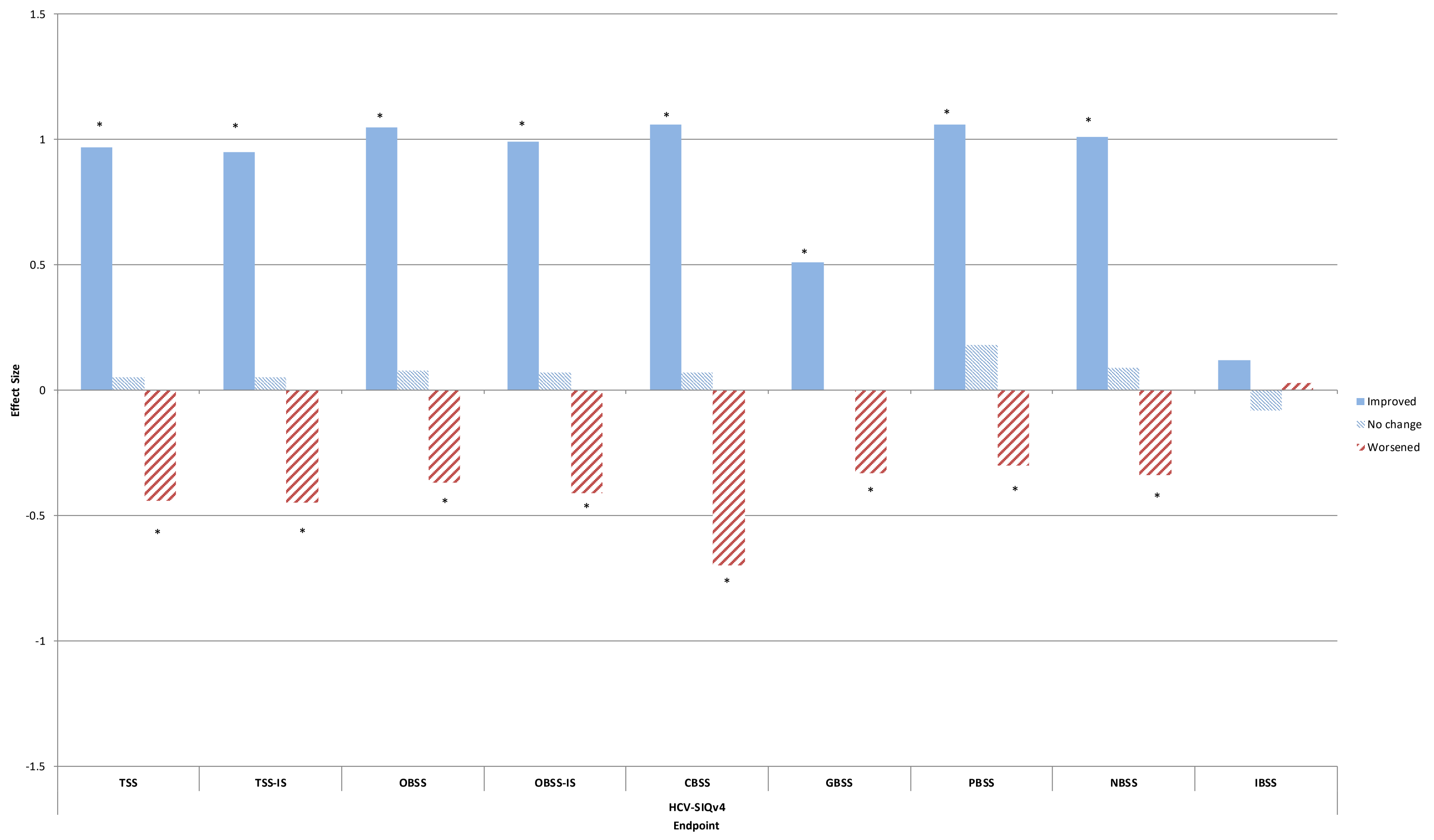

* Denotes p<0.05 as assessed by two-sample t-test between groups

Supplemental Figure 4. Responsiveness of HCV-SIQv4 Scores from Baseline to Last Study Visit, by Adverse Events over this Time

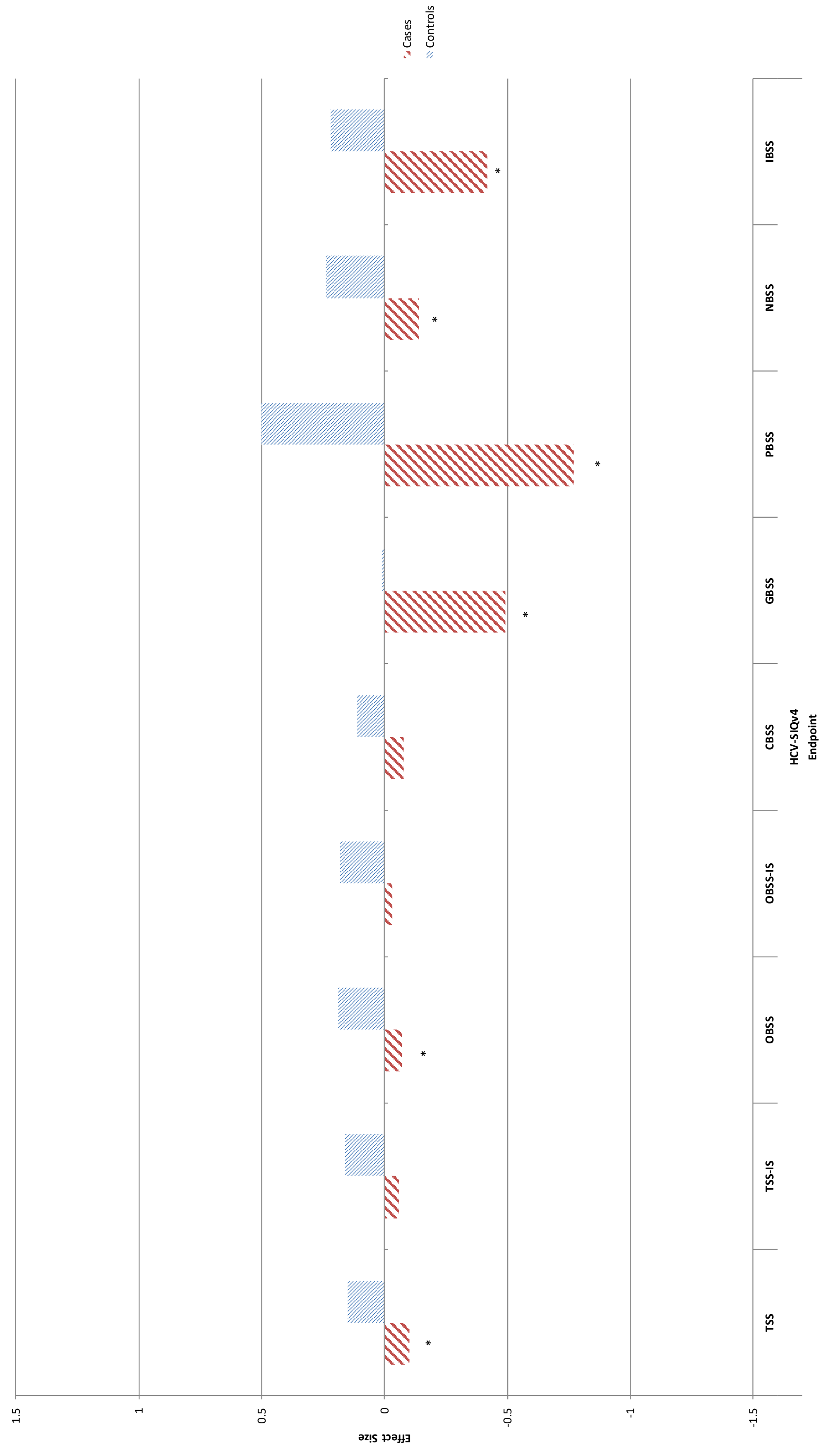

* Denotes $p<0.05$ as assessed by two-sample t-test between groups

Supplemental Figure 5. Responsiveness of HCV-SIQv4 Scores from Baseline to Last Study Visit, by change in SVR12 over this Time

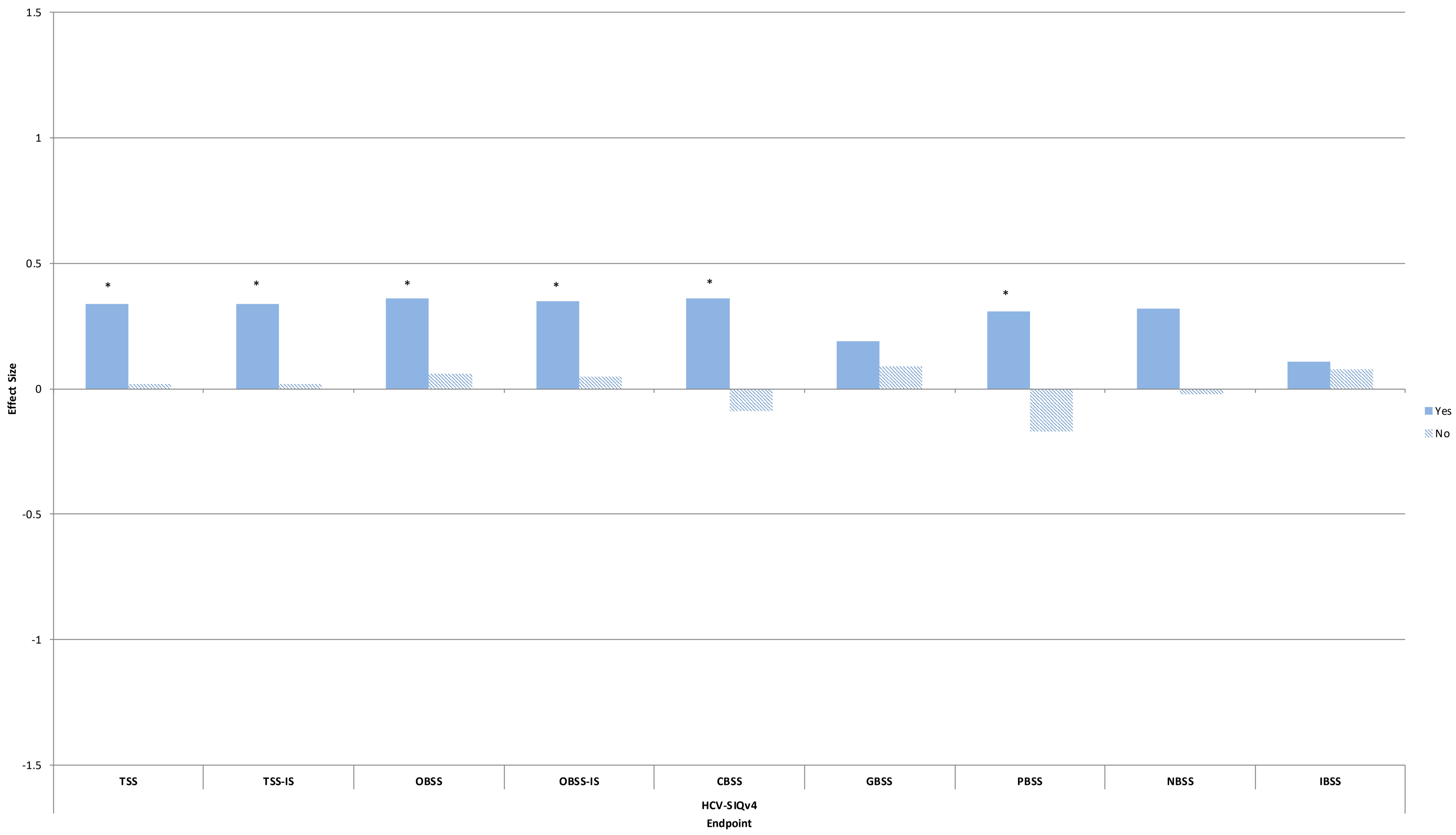

* Denotes p<0.05 as assessed by two-sample t-test between groups

Supplemental Figure 6. Responsiveness of HCV-SIQv4 Scores from Baseline to Last Study Visit, by Viral Relapse over this Time

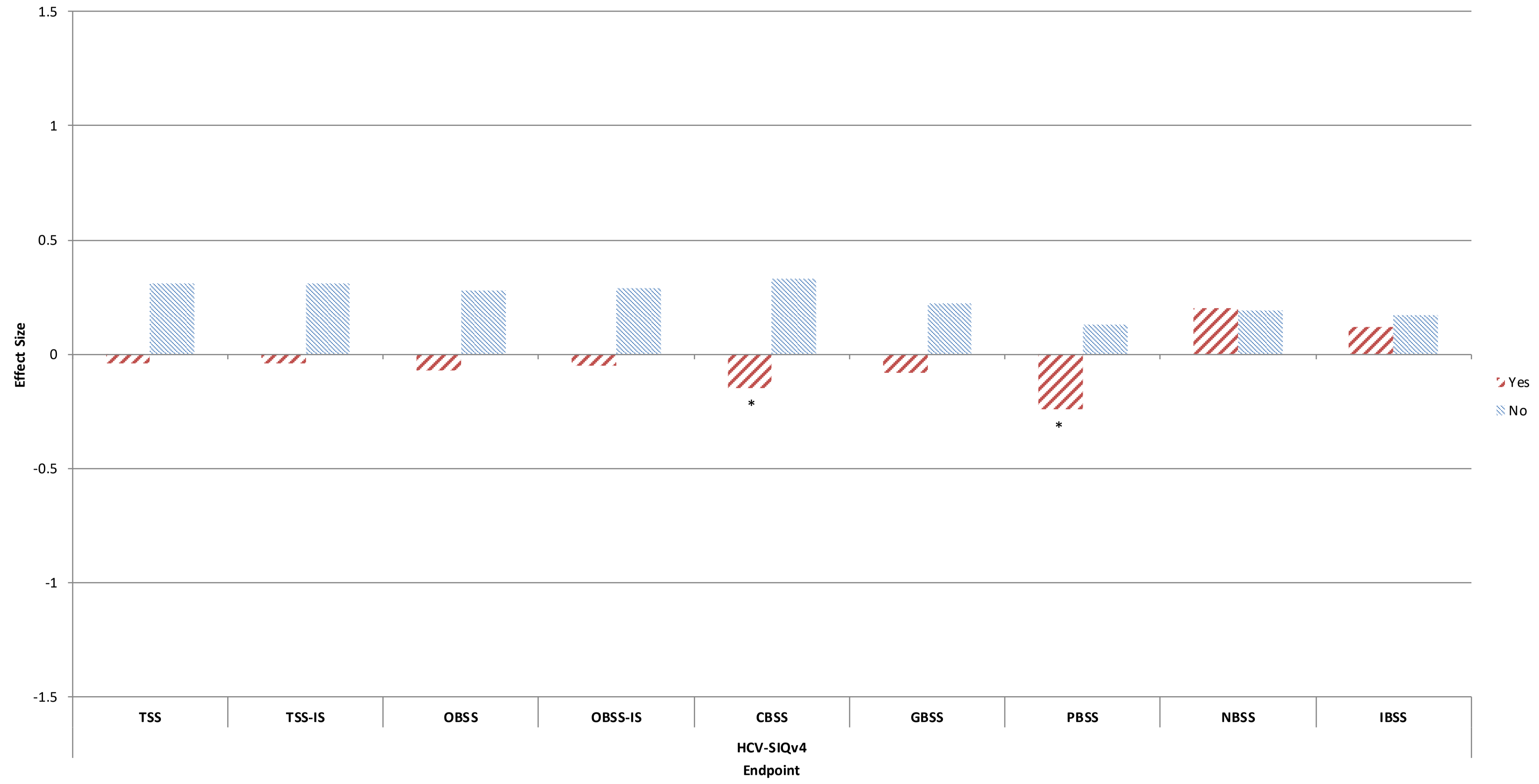

* Denotes $p<0.05$ as assessed by two-sample t-test between groups
